# Novel Yttria-Stabilized Zirconium Oxide and Lithium Disilicate Coatings on Titanium Alloy Substrate for Implant Abutments and Biomedical Application

**DOI:** 10.3390/ma13092070

**Published:** 2020-04-30

**Authors:** Julius Maminskas, Jurgis Pilipavicius, Edvinas Staisiunas, Gytis Baranovas, Milda Alksne, Povilas Daugela, Gintaras Juodzbalys

**Affiliations:** 1 Department of Prosthodontics, Lithuanian University of Health Sciences, 50106 Kaunas, Lithuania; 2 Department of Chemical Engineering and Technology, Center for Physical Sciences and Technology (FTMC), 02300 Vilnius, Lithuania; jurgis.pilipavicius@ftmc.lt; 3 Faculty of Chemistry and Geosciences, Vilnius University, 10257 Vilnius, Lithuania; edvinas.staisiunas@chf.stud.vu.lt (E.S.); gytis.baranovas@chf.stud.vu.lt (G.B.); 4 Institute of Biochemistry, Life Sciences Center, Vilnius University, 10257 Vilnius, Lithuania; milda.peciukaityte@gf.vu.lt; 5 Department of Maxillofacial Surgery, Lithuanian University of Health Sciences, 50140 Kaunas, Lithuania; povilas.daugela@lsmuni.lt (P.D.); gintaras.juodzbalys@lsmuni.lt (G.J.)

**Keywords:** coatings, focal adhesions, implant abutments, roughness, zirconium oxide

## Abstract

This study aimed to create novel bioceramic coatings on a titanium alloy and evaluate their surface properties in comparison with conventional prosthetic materials. The highly polished titanium alloy Ti6Al4V (Ti) was used as a substrate for yttria-stabilized zirconium oxide (3YSZ) and lithium disilicate (LS2) coatings. They were generated using sol-gel strategies. In comparison, highly polished surfaces of Ti, yttria-stabilized zirconium oxide (ZrO_2_), polyether ether ketone (PEEK) composite, and poly(methyl methacrylate) (PMMA) were utilized. Novel coatings were characterized by an X-ray diffractometer (XRD) and scanning electron microscope (SEM). The roughness by atomic force microscope (AFM), water contact angle (WCA), and surface free energy (SFE) were determined. Additionally, biocompatibility and human gingival fibroblast (HGF) adhesion processes (using a confocal laser scanning microscope (CLSM)) were observed. The deposition of 3YSZ and LS2 coatings changed the physicochemical properties of the Ti. Both coatings were biocompatible, while Ti-3YSZ demonstrated the most significant cell area of 2630 μm^2^ (*p* ≤ 0.05) and the significantly highest, 66.75 ± 4.91, focal adhesions (FAs) per cell after 24 h (*p* ≤ 0.05). By contrast, PEEK and PMMA demonstrated the highest roughness and WCA and the lowest results for cellular response. Thus, Ti-3YSZ and Ti-LS2 surfaces might be promising for biomedical applications.

## 1. Introduction

Stable and healthy surrounding tissues define the success of a dental implant [1,2]. The importance of bone tissue is not questionable from a biomechanical point of view [3,4,5], but the soft tissue is of biological significance [6]. Currently, the prevention of peri-implant diseases is attracting increasing attention, and according to tendencies of the latest studies, the relevance of soft tissue is becoming more popular [7,8]. The function of the soft tissue barrier is protective and prevents infectious pathogens from entering and spreading, so one of the essential criteria for implant success is peri-implant soft tissue sealing [9]. Moreover, soft tissue acts not only as a physical but also as a biological barrier, which has immune response capacity against microorganisms [10,11].

Peri-implant soft tissue is composed of epithelium cells and connective tissue, which is composed of human gingival fibroblasts (HGFs) [12]. They have several functions, such as immune response and secretion of extracellular matrix (ECM) proteins [13]. The collagen expressed by fibroblasts is especially important in forming the peri-implant soft tissue structure because it determines the firmness of the gingiva. Moreover, the alignment of collagen fibers influences the stability of soft tissue sealing.

The gingiva around the implant abutment, similar to the tooth, forms the biological width, and the implant is sealed by the epithelial junction above [14]. However, comparing the tooth with the implants, the main differences exist because of artificial materials and their modulation of gingival cell behavior [15]. It is believed that the soft tissue architecture around the implant abutment may be determined by the physical and chemical properties of the interfaced surface [16]. Properties such as surface chemical composition, surface hydrophilicity, surface free energy (SFE), or roughness may alter cellular vitality, cellular adhesion, cellular type, and collagen fiber concentration and arrangement [17]. Feedback interactions link surrounding cells with the ECM, which is formed by proteins and covers an implanted surface [18]. It communicates with the cell through their transmembrane receptors: integrins. While extracellular integrin domains interact with ECM proteins, the intracellular receptor parts together with various cellular proteins (such as vinculin, paxillin, talin, etc.) form macromolecular structures: focal adhesions (FAs). FAs determine anchorage, coupling mechanical and chemical signaling, force generation, and mechanosensing of cells [19]. The number of FAs indicates the strength of cell adhesion on the surface, while its area describes the FA contact. Additionally, the amount of focal adhesions (FAs) is related to the substrate surface, because it can affect cellular traction forces, causing a reorganization of the cell’s cytoskeleton [20]. Because the restorative material should not only be biocompatible itself but also achieve an epithelial seal and ensure surface properties for biocompatibility and cell adhesion, it is important to quantify the cell adhesion strength on these kinds of materials [21].

On the other hand, surface properties, such as morphology correlate with bacterial adhesion [22] and it should not be forgotten that the surface of restorative materials must be unfavorable for plaque accumulation because it is the bacteria that cause the peri-implant mucositis [8,23]. Despite proper soft-tissue sealing, the bacteria may disturb the barrier by producing virulence factors and go down the implant [24,25].

Therefore, the surface of artificial materials at a transmucosal level should be unfavorable for bacteria attachment as well as bioinert and favorable for soft-tissue adhesion and vitality of cells [26,27]. Bacteria and cells differ not only in size and morphology but also in adhesion mechanisms, so it is essential to understand which surface properties of the materials should be achieved [28].

Nowadays, there exist a variety of surface treatments, such as mechanical treatments, laser treatment, chemical activation, plasma treatment, or coatings [29]. Notably, the trend of coatings is growing, because thin functional layers of organic and inorganic additives can be spread with purposes of soft-tissue engineering. Undoubtedly, these methods change surface properties, which are essential for interaction with host cells as well as with bacterial strains, and surface improvements might be one of the peri-implant disease prevention possibilities. Thus, to improve usual implant abutment surfaces, we decided to modify the polished surface of titanium alloy (Ti6Al4V) using sol-gel derived bioceramic coatings.

The aim of this study was to create novel experimental bioceramic coatings on the titanium alloy and evaluate their surface physicochemical and biological properties in comparison with conventional dental implant prosthetic materials after polishing.

## 2. Materials and Methods

### 2.1. Specimen Preparation

The plate shape of 10 × 10 × 0.5 mm^3^ specimens was used for this study. They were milled using the computer-aided design and computer-aided manufacturing system (Dental Concept Systems DC1, Dental Concept Systems GmbH, Ulm, Germany) from commercially available prosthetic materials. Four groups were prepared (*n* = 10): titanium alloy Ti6Al4V (Ti) (Ti6Al4V, DC Titan 5, Dental Concept Systems GmbH, Ulm, Germany); yttria-stabilized zirconium oxide (ZrO_2_) (ZrO_2_ 3Y-TZP Nacera Pearl, Doceram Medical Ceramics GmbH, Dortmund, Germany); polyether ether ketone composite (PEEK) (BioHPP, Bredent GmbH, Senden, Germany); and poly(methyl methacrylate) (PMMA) (Brecam Universal, Bredent GmbH, Senden, Germany). Samples of the ZrO_2_ group were additionally sintered in the furnace (Zubler Vario S400, Zubler USA, Dallas, TX, USA) for 2 h at 1450 °C. Additionally, milled raw specimens of the polished titanium alloy were used for two experimental groups of coatings as substrates: yttria-stabilized zirconium oxide coating (3YSZ) on Ti substrate (Ti-3YSZ) and lithium disilicate coating (LS2) on Ti substrate (Ti-LS2). 

### 2.2. Surface Preparation

To achieve identical conditions, polishing was performed using a rotary machine (Holzmann metal lathe ED3000ECO, Maschinenhandel Gronau Inc., Gehren, Germany). The surface of all specimens was polished with decreasing coarseness of water-resistant silicon carbide (SiC) abrasive paper P2000, P2500, P3000, P4000 (Starcke GmbH and Co. KG, Melle, Germany) and later with SiC abrasive pad P5000 (Trizact™, 3M Company, St. Paul, MN, USA) under water cooling/washing. The final polishing was completed with a diamond polishing paste and a natural brush (Zirkopol, Feguramed GmbH, Odenwald, Germany). The polishing cycle for each coarseness was performed for 60 s at 3000 rpm.

### 2.3. Reagents for Coatings

Yttrium nitrate hexahydrate (99%) and acetylacetone (AcAc, 99.5%) were purchased from Sigma-Aldrich Inc. (St. Louis, MO, USA); zirconium propoxide (ZIP, 70% sol. in 1-propanol), lithium methoxide (LiOMe, 2.2M sol. in methanol), and tetramethyl orthosilicate (TMOS 99%) were purchased from Acros Organics (Acros Organics™, Thermo Fisher Scientific, Waltham, MA, USA). All reagents were used as received. Methanol, ethanol, and isopropanol were kept on 3A molecular sieves for 48 h and further distilled under dry nitrogen.

### 2.4. Preparation of Stabilized Zirconium Oxide (3YSZ) and Lithium Disilicate (LS2) Sols

The coatings were prepared using the sol-gel process, initially preparing an alkoxide solution of the precursors, followed by spin-coating them on the polished titanium alloy substrate. For the preparation of 3YSZ (by mol% Y_2_O_3_) sol, 0.0073 mol of yttrium nitrate hexahydrate and 0.115 mol of ZIP were dissolved in dry 2-propanol solution stabilized with 0.0345 mol of AcAc. To initiate a hydrolysis reaction, 0.23 mol of H_2_O was added in a final step. 

For the preparation of lithium disilicate, LiOMe sol 0.01 mol and 0.01 mol TMOS were dissolved in dry methanol, then 0.015 mol of H_2_O was added in a final step. Both solutions were kept at room temperature (RT) for 24 h, and after that, obtained sols were ready for the spin-coating procedure.

### 2.5. Deposition of 3YSZ and LS2 Coatings

Prior to the coating procedure, prepared sols were filtered through a 0.2 µm nylon membrane filter. Ti substrates were cleaned with a non-ionic surfactant solution (RBS Neutral T, Carl-Roth) in an ultrasonic bath (Sonorex, BANDELIN electronic GmbH and Co., Berlin, Germany), then thoroughly washed with deionized H_2_O and 2-propanol and allowed to dry in air. To prepare the desired coating, 50 µL of the corresponding sol was placed on a Ti substrate and spin-coated up to 2000 rpm. Prepared 3YSZ and LS2 coatings were placed in a muffle furnace (SNOL 13/1100, Umega group, Utena, Lithuania) and heated at 600 °C for 2 h. After this step, prepared coatings were evaluated using X-ray diffraction (XRD) and ready for other experiments. A commercial X-ray diffractometer (Rigaku SmartLab, Rigaku Corporation, Tokyo, Japan) was used for the XRD analysis of deposited coatings. To achieve higher sensitivity, spectra were recorded by employing grazing angle geometry, 0.01 deg. step size, and 1 deg/min scan speed. For evaluation of coating thickness, several coatings were applied to a 10 × 10 mm silicon (100) wafer. All coating procedures were the same as for the Ti. 

### 2.6. Surface Morphology

Surface morphology and roughness parameters of specimens were characterized by using a scanning electron microscope (SEM) (Hitachi SU-70, Hitachi, Ltd., Chiyoda, Tokyo, Japan) and atomic force microscope (AFM) (Agilent 5500 AFM/SPM, Agilent Technologies, Palo Alto, CA, USA). Data on the means of the surface topography and roughness average (S_a_) were collected by randomly scanning each sample in three areas. AFM topographic images were recorded in contact mode using a silicon probe with a 10 nm tip radius. Each image was recorded in 256 × 256 pixel size. For evaluation of coating thickness, Si-coated samples were broken in half, and the thickness of sample cross-sections was measured by SEM.

### 2.7. Water Contact Angle and Surface Free Energy

Specimens were all cleaned in 2-propanol and bi-distilled water using sonication. They were then dried for 2 h in a vacuum oven at 50 °C. An optical tensiometer (CAM 200, KSV Instruments Ltd., Helsinki, Finland) was used to measure the WCA and SFE. For measurement purposes, bi-distilled water was used in a heavy phase. To measure the WCA of the solvent droplet, a modest drop (4–7 µL) of a particular liquid was placed on a tested substrate at RT. The SFE was calculated using the Owens-Wendt method [30].

### 2.8. Cell Culture

Human gingival fibroblasts (HGF-1; CRL-2014, ATCC) were retained in Dulbecco’s Modified Eagle Medium (DMEM) combined with 10% fetal bovine serum (FBS) (Gibco, Thermo Fisher Scientific, Waltham, MA, USA) and a penicillin (100 U/mL), streptomycin (100 mg/mL) antibiotics solution (Gibco, Thermo Fisher Scientific, Waltham, MA, USA). It was completed at 37 °C in a humidified atmosphere containing 5% CO_2_ until 70–80% confluence was achieved. The cells used in the experiments were up to 15 passages.

### 2.9. Biocompatibility Evaluation

To determine the biocompatibility of specimens, 3-[4,5-dimethylthiazole-2-yl]-2,5-diphenyltetrazolium bromide (MTT) assay was used. It was conducted by seeding HGF-1s (3 × 10^4^ cell/cm^2^) in tissue culture plates. After 24 h, samples were placed on an HGF-1 monolayer and were incubated for a further 24 h. After reaching a predetermined time point, the growth media was removed, and specimens were rinsed once with phosphate-buffered saline (PBS). Furthermore, in each sample, 0.2 mg/mL MTT (MTT, Sigma-Aldrich Inc., St. Louis, MO, USA) solution was added in PBS and incubated for 1 h at 37 °C in a humidified atmosphere consisting of 5% CO_2._ MTT solution was subsequently discarded, and formed formazan was dissolved in dimethyl sulfoxide (DMSO) (dimethyl sulfoxide, Sigma Aldrich, Dorset, UK) by incubating with mild shaking (25 rpm) at RT for 10 min. Using a microplate spectrophotometer (Varioskan Flash, Thermo Fisher Scientific, Inc., Waltham, MA, USA, the absorbance was evaluated at 570 nm. A specimen without contact with cells was attributed as a background value.

### 2.10. Cellular Adhesion

A quantitative and qualitative assessment was used to determine cell adhesion efficiency and adhesion strength on tested specimens by applying F-actin staining and FA visualization. To achieve this, HGF-1s were cultivated (15 × 10^3^ cell/cm^2^) for 2 and 24 h on tested surfaces. After specific time points that were previously determined, specimens were fixed with 4% paraformaldehyde (Carl Roth, GmbH, Karlsruhe, Germany) in PBS (Gibco, Thermo Fisher Scientific, Waltham, MA, USA). This was achieved at RT for 15 min with mild agitation (25 rpm). Samples were then rinsed twice in PBS with 0.05% Tween-20 (Sigma-Aldrich Co., St. Louis, MO, USA), permeabilized with 0.2% Triton X-100 (Sigma-Aldrich Co., St. Louis, MO, USA) in PBS for 5 min at RT with 25 rpm agitation, and then washed twice with 0.05% Tween-20 solution. After that, specimens were blocked for 30 min with 3% bovine serum albumin (BSA; AppliChem GmbH, Darmstadt, Germany) and 10% FBS prepared in PBS. After the subsequent blocking procedure, samples were incubated with primary mouse anti-vinculin antibody (1:50; Merck Millipore, Burlington, MA, USA) at RT for 1 h in blocking solution while using gentle shaking at 25 rpm. Soon after, specimens were washed three times for 5 min with a 0.05% Tween-20 solution. They were then incubated with secondary goat anti-mouse Alexa Fluor 488-conjugated antibodies (Invitrogen, Carlsbad, CA, USA) and tetramethyl rhodamine iso-thiocyanate (TRITC)-conjugated phalloidin (1:500; Merck Millipore, Carlsbad, CA, USA) in PBS in a darkened environment at RT for 1 h with 25 rpm agitation. After that, the samples were washed three more times with PBS for 5 min at RT and stained with 12.5 µg/mL 4’,6-diamidino-2-phenylindole (DAPI; Merck Millipore, Carlsbad, CA, USA) solution in PBS for 5 min in the dark at RT with 25 rpm agitation. Then, specimens were washed three times with PBS for 5 min at RT and visualized using a confocal laser scanning microscope (CLSM) (Leica SP5 TCS, Leica Microsystems, Wetlzer, Germany). Using the image processing program ImageJ (1.8.0_112) (Wayne Rasband, National Institute of Mental Health, Bethesda, MD, USA), measurement of the cells’ surface area and counting of FAs within the cells on different samples were performed. Then, the quantitative differences in cells’ adhesion efficiency and strength were determined.

### 2.11. Statistical Analysis

The specimen size was calculated using G*Power 3.1 software (Heinrich Heine University, Dusseldorf, Germany) before the study. The data were processed using the GraphPad Prism 8 software package (San Diego, CA, USA). The roughness, WCA, SFE, relative HGF-1 cell counts, and adhesion area measurements were analyzed based on standard deviations and means. All data sets were checked for their normality using the D’Agostino-Pearson omnibus test and then analyzed by one-way analysis of variance (ANOVA) followed by Tukey’s multiple comparison test. Additionally, the relationship between experiments was evaluated based on Pearson correlations. A *p*-value of ≤0.05 was considered statistically significant. 

## 3. Results

### 3.1. Phase Composition

**Figure 1:**
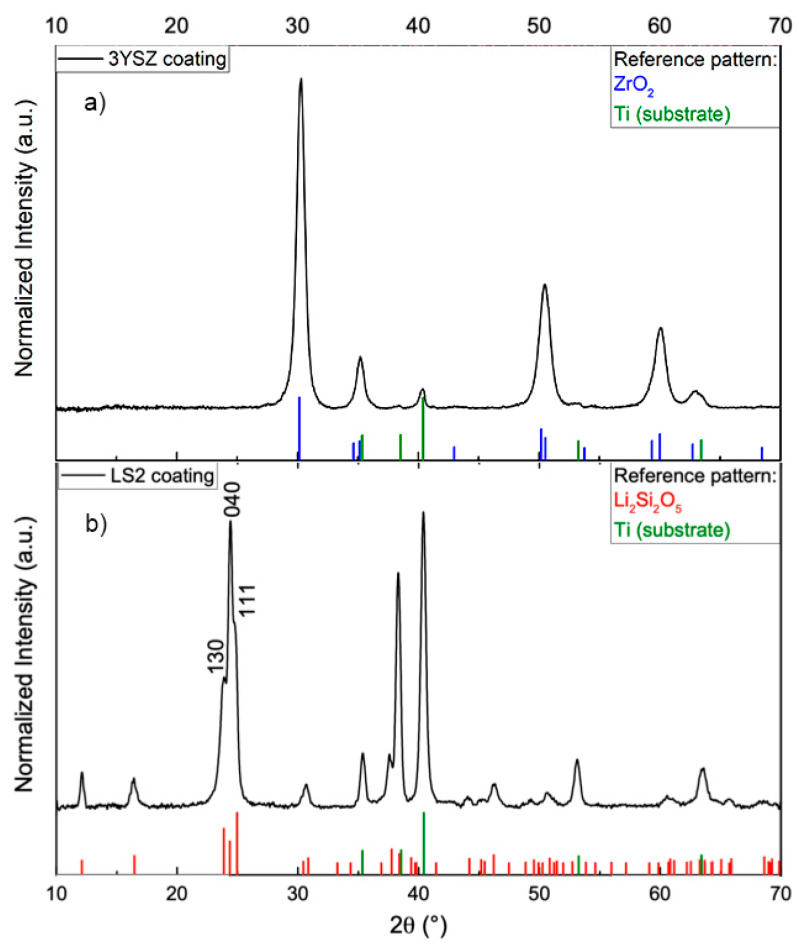
X-ray diffraction (XRD) spectra of coated surfaces: (**a**) yttria-stabilized zirconium oxide coating on titanium alloy (Ti-3YSZ); (**b**) lithium disilicate coating on titanium alloy (Ti-LS2).

The formation of ceramic 3YSZ and LS2 glass-ceramic coatings was confirmed by XRD (Figure 1). In both cases, single-phase crystalline coatings were obtained. Some additional diffraction peaks of the Ti alloy substrate were visible in both patterns. In the case of the 3YSZ coating, observable peaks in the XRD pattern correspond to the tetragonal ZrO_2_ phase. Visible diffraction peaks on XRD patterns of the LS2 coating can be associated with the orthorhombic lithium disilicate phase. The LiSiO_3_ phase commonly found in lithium silicate glass-ceramics was not detected in this case. It is important to note that the diffraction peak on LS2-coated Ti corresponding to the (040) plane was more intense than expected, which could indicate the formation of slightly texturized lithium disilicate crystal orientation. These findings on both surfaces established that coatings of monophase 3YSZ and LS2 were achieved by the sol-gel method. Other groups were excluded due to lack of chemical modification of polished surfaces.

### 3.2. Surface Characterization

The surface roughness of each group was measured after preparation. The S_a_ means were used to express the measurements. The S_a_of all surfaces was below the micro level (Table 1). After polishing, the lowest roughness was found on the ZrO_2_ surface (S_a_ = 5.53 ± 0.21 nm), and the highest on PMMA (S_a_ = 65.23 ± 2.41 nm). S_a_ values were significantly different between all groups (*p* < 0.0001) (Table 2). The coatings of both bioceramics reduced the roughness of the polished titanium alloy substrate surface. Compared to the surface of the polished titanium alloy (S_a_ = 17.67 ± 0.35 nm), after the deposition of the 3YSZ coating (S_a_ = 16.61 ± 0.52 nm), the decrease in surface roughness was not statistically significant (*p* > 0.05), but the LS2 coating (S_a_ = 9.61 ± 0.66 nm) decreased the roughness significantly (*p* < 0.0001).

**Figure 2:**
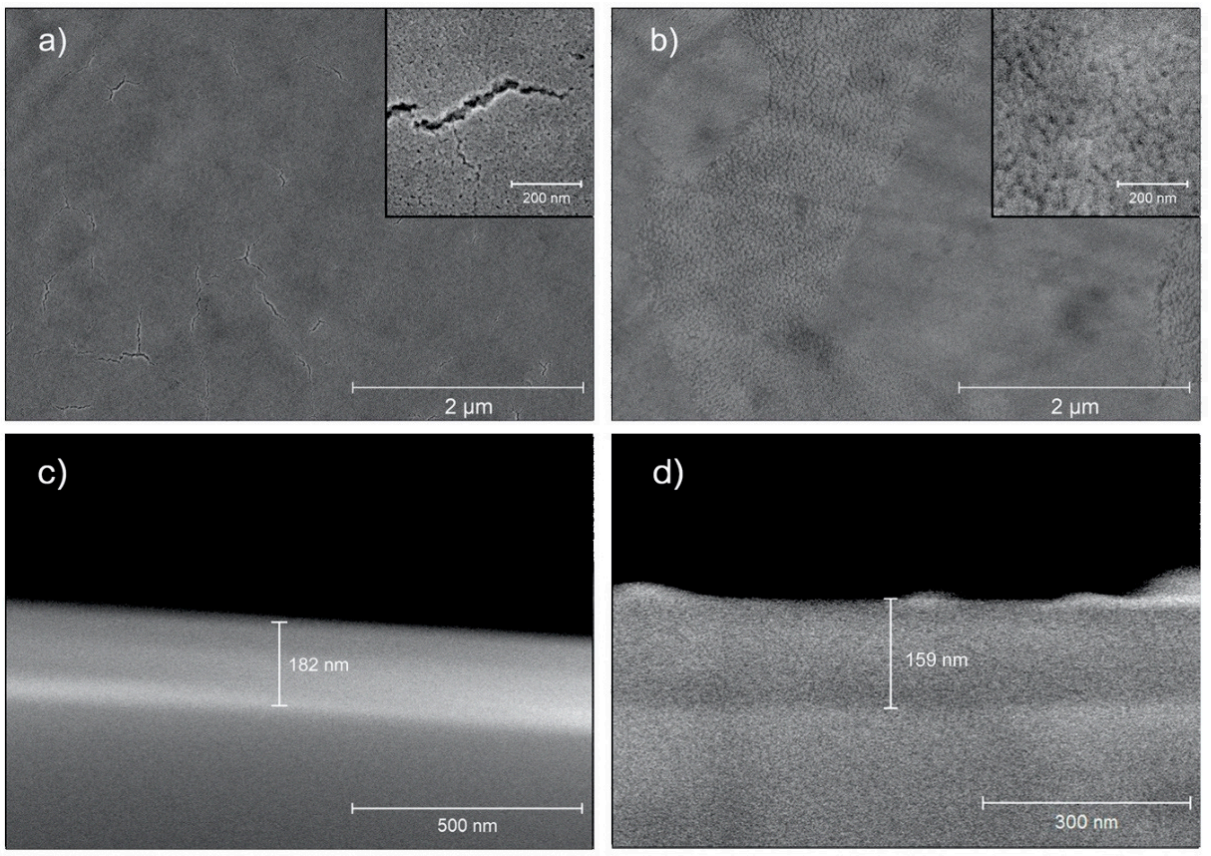
Scanning electron microscope (SEM) pictures of nanocoated surfaces: (**a**) Ti-3YSZ; (**b**) Ti-LS2; (**c**) cross-section of 3YSZ coating; (**d**) cross-section of LS2 coating.

The morphology of bioceramic coatings in Ti-3YSZ and Ti-LS2 was evaluated by SEM. In both images, a uniform surface structure was observed. In the image (Figure 2a), small cracks in the 3YSZ coating were detected, while the LS2 layer was without cracks and regular (Figure 2b). All detected cracks were less than 1 µm in size. 3YSZ coatings consist of a uniform layer of nano-sized grains, whereas LS2 contains micron-sized domains of differently orientated nano-sized grains and/or glassy phase. The grain size of both ceramic coatings was less than 10 nm. The determination of the exact crystallite size was limited by instrument resolution. The determined coating thickness of 3YSZ and LS2 was 182 nm and 159 nm, respectively (Figure 2c,d).

### 3.3. Water Contact Angle and Surface Free Energy

All polished surfaces of commercially available abutment materials demonstrated relatively high WCA (Table 3), which means higher hydrophobicity. The WCA means of all groups were significantly different (*p* ≤ 0.05). The lowest difference was between the Ti (60.21 ± 1.58°) and ZrO_2_ (57.32 ± 1.86°) groups (*p* = 0.0264), while the difference among the remaining groups was even higher (*p* < 0.0001). WCA measurements established the influence of coatings because of this increased hydrophilicity of the titanium alloy surface. The WCA values of Ti-3YSZ (32.58 ± 1.94°) and Ti-LS2 (25.71 ± 1.33°) were statistically lower than the Ti alloy itself (60.21 ± 1.58°), as well as others (*p* < 0.0001). The highest hydrophilicity was detected in the Ti-LS2 group.

Separate SFE data were collected, investigating polar and dispersive SFE (Table 1). The total SFE was the sum of these parts. The Ti-3YSZ (62.03 ± 0.57 mN/m) and Ti-LS2 (65.70 ± 1.00 mN/m) coatings significantly increased total SFE values compared to the Ti alloy (41.81 ± 2.01 mN/m) (*p* < 0.0001) (Table 4), while all conventional prosthetic materials showed lower and similar total SFE values. The PEEK showed an extra low value of polar SFE (4.14 ± 0.2 mN/m), while Ti-LS2 showed the highest polar part (33.39 ± 0.59 mN/m) compared to groups with intermediate values (Table 1). Moreover, the SFE polar component was higher in both coated groups (Table S1). The highest dispersive SFE was determined on the PEEK surface (39.32 ± 0.38 mN/m), while the lowest was on the ZrO_2_ (30.38 ± 0.55 mN/m). A significant difference of means was observed among all groups (*p* < 0.0001), except Ti-3YSZ (36.89 ± 0.33 mN/m) and PMMA (36.98 ± 0.59 mN/m) groups (*p* = 0.9998) (Table S2). A strong negative correlation between WCA and total SFE was established (r = −0.9348) (Table S3).

### 3.4. Biocompatibility Evaluation

**Figure 3:**
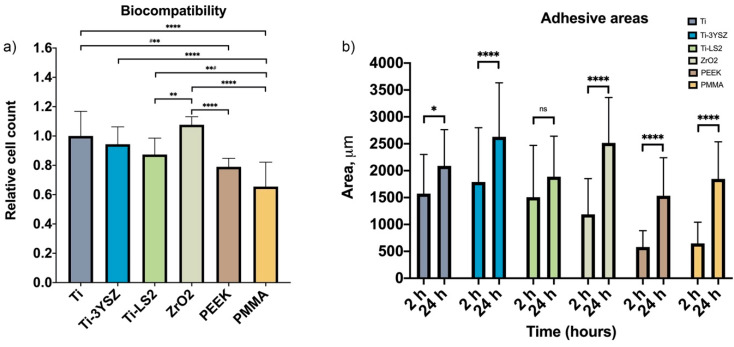
(**a**) Charts of biocompatibility expressed by means and standard deviations of human gingival fibroblast (HGF) relative cell counts (*n* = 10 for each group). Multiple comparisons by Tukey’s test and significant difference at the following levels: **** *p* < 0.0001; ^#^** *p* = 0.004; ** *p* = 0.006; **^#^ *p* = 0.0024; ns *p* > 0.05 (for all unmarked groups). (**b**) Adhesive areas (μm) of HGF-1-stained actin on surfaces at a different times (2 and 24 h) expressed by means and standard deviations. Multiple comparisons of different times in groups by Tukey’s test and significant difference at the following levels: **** *p* < 0.0001; * *p* = 0.0232; ns *p* > 0.05.

The biocompatibility of the newly formed coatings on the Ti alloy surface was evaluated and compared with the biocompatibility of conventional implant prosthetic materials. The results were standardized by the number of cells obtained on the polished Ti alloy control surface. After evaluating the growth of relative HGF-1 cell count (RCC), it was found that all materials used for the assay were biocompatible (Figure 3a). The highest RCC (i.e., the best biocompatibility) was observed for the ZrO_2_ (1.08 ± 0.05) surfaces. Moreover, no statistically significant differences were found between polished Ti (1.00 ± 0.17) and Ti-3YSZ (0.94 ± 0.12) (*p* > 0.05) or Ti (1.00 ± 0.17) and Ti-LS2 (0.87 ± 0.11) (*p* > 0.05) specimens. The lowest biocompatibility was found in the PMMA (0.65 ± 0.11) group, which was significantly different from others (*p* < 0.0001), except PEEK (0.79 ± 0.06) (*p* > 0.05).

### 3.5. Cell Adhesion Area

**Figure 4:**
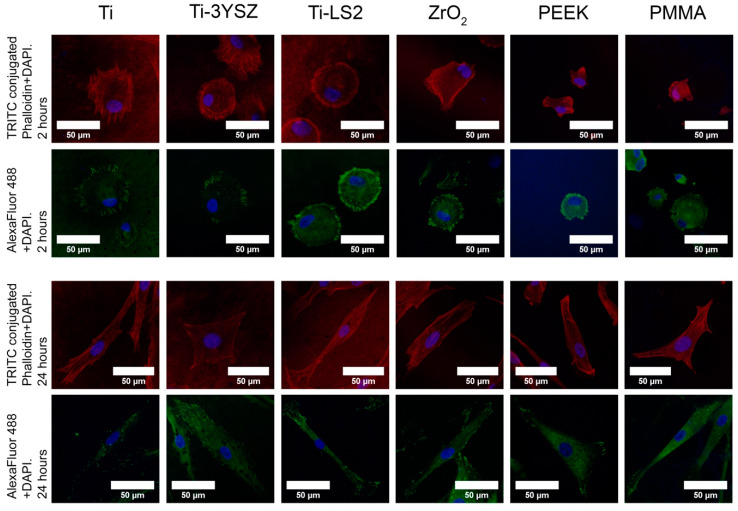
Confocal laser scanning microscope (CLSM) images of immunohistochemically stained cells on surface coatings at a different time (2 and 24 h). HGF-1 nucleus (DAPI (4’,6-diamidino-2-phenylindole), blue), F-actin filaments (tetramethyl rhodamine iso-thiocyanate (TRITC)-conjugated phalloidin, red), and focal adhesion (FA) spots (vinculin stained with Alexa Fluor 488-conjugated antibodies, green).

HGF-1 adhesion on tested specimens was determined by calculating cell surface areas 2 and 24 h post-seeding. The HGF-1 surface area was visualized by cell F-actin staining. To evaluate cell adhesion differences that occurred on tested specimens, a qualitative and quantitative assessment of the images obtained by CLSM was performed. The results of HGF-1 adhesion showed that all tested surfaces were attractive for cell attachment. Changes in the HGF-1 surface area (2 and 24 h after seeding) showed that the cells were growing on all specimens, as the surface areas increased during the time (Figure 3b and Figure 4).

Moreover, 2 h after seeding, the largest HGF-1 surface area (the best cell adhesion) was found on the Ti-3YSZ specimens (1789 μm^2^) (*p* < 0.0001), and the lowest cell area was determined on both polymeric surfaces (PEEK (581.8 μm^2^) and PMMA (648.6 μm^2^), but these two were not significantly different. Furthermore, after 24 h, the highest cell adhesion area was observed on the Ti-3YSZ (2630 μm^2^) specimens, and the lowest was determined on polymeric PEEK (1534 μm^2^) and PMMA (1847 μm^2^) surfaces as well as Ti-LS2 specimens (1888 μm^2^).

It is also important to mention that compared to 2 h, after 24 h, the cell attachment area increased in all groups except the Ti-LS2 group (1507 μm^2^ after 2 h and 1888 μm^2^ after 24 h), where the increment of adhesive cell area was not statistically significant (*p* > 0.05). Moreover, even though the increase of cell surface area on PEEK and PMMA samples after 24 h was statistically significant (*p* < 0.0001), it reached only a similar surface area size as on Ti-3YSZ after 2 h (Figure 3b).

### 3.6. Cell Focal Adhesion (Density Distributions)

**Figure 5:**
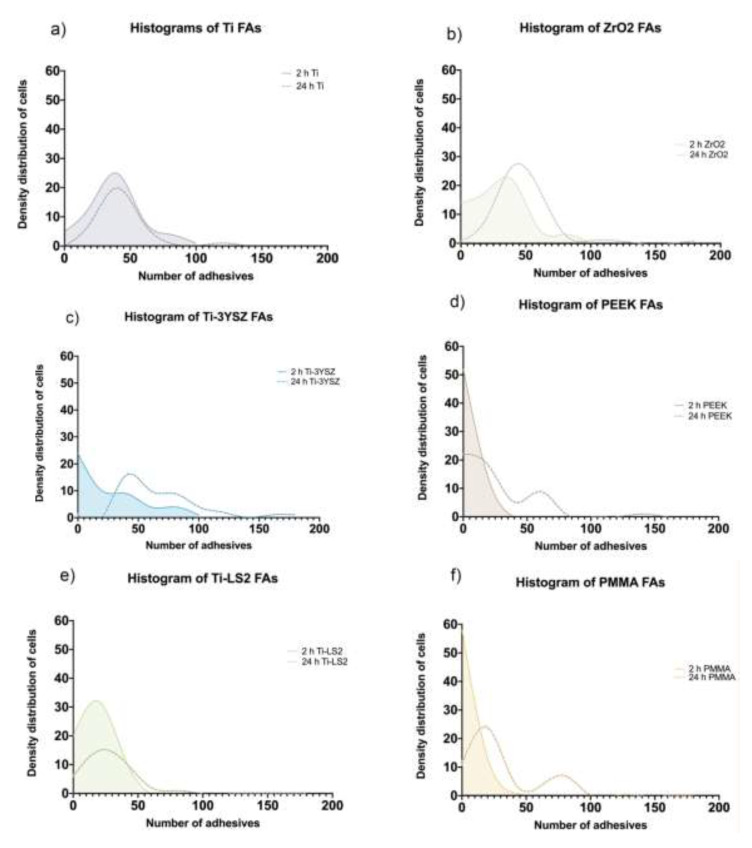
Histograms of HGF focal adhesions (FAs) at a different time 2 (line + fill) and 24 h (dashed line) after seeding: (**a**) Ti; (**b**) ZrO_2_; (**c**) Ti-3YSZ; (**d**) PEEK; (**e**) Ti-LS2; (**f**) PMMA.

Immunohistochemically stained FAs were visualized (Figure 4) and quantified (Figure 5) to evaluate cell adhesion strength. Obtained quantitative FA results were expressed as the density distribution function of cells and number of focal adhesions 2 and 24 h after seeding. Also, means of FAs per cell were provided for statistical data comparison (Tables S4 and S5 and Table 5).

The images obtained by CLSM showed that 2 h after seeding, cells began to form FAs on all examined surfaces (Figure 4); moreover, the quantitative results revealed the differences between them (Figure 5). It was observed that on polymeric PEEK and PMMA surfaces, most of the cells did not form FAs (Figure 5d,f) or formed only a small amount of them: PEEK—5.15 ± 0.74 FAs/cell, PMMA—5.76 ± 0.93 FAs/cell. The projections of both histograms had similar configurations and revealed that the surfaces of both polymers were not attractive for an early cell attachment. A wide range of FA distribution was observed on the Ti-3YSZ surface (Figure 5c). In this group, cells were relatively uniformly distributed with a similar quantity of 0 to 100 FAs. Additionally, the concentration of cells without FAs on Ti-3YSZ was lower than on polymers but higher than on other surfaces, while cells with the highest FA counts after 2 h were also the highest. No trend in cellular concentration by FAs was observed in this group, while its mean was 28.00 ± 3.76 FAs/cell. In the remaining groups, FA numbers in cells increased respectively: Ti-LS2 FAs ranged from 15 to 20 (Figure 5e), ZrO_2_ FAs ~35 (Figure 5b), and Ti FAs ~50 (Figure 5a). However, both the ZrO_2_ and Ti alloy histograms replicated the portion of the Ti-3YSZ plot showing similar concentrations of the most FA-rich cells, and the concentration of the FAs within the cells in all three groups were very similar. Comparing FAs/cell, the statistical difference did not exist between these three groups (*p* > 0.05) (Table S4).

Twenty four-hour results revealed that the highest number of FAs were formed in HGF-1 grown on the Ti-3YSZ surface (Figure 5c). Two peaks of cell concentrations were detected in this group. The highest cell concentration was ~45 FAs per cell, but even a wave of 75–80 FAs per cell was observed. The mean on Ti-3YSZ group was 66.75 ± 4.91 FAs/cell, and it was statistically the highest (*p* ≤ 0.05) (Table S5). By contrast, the PEEK group showed persistent high concentrations of cells that formed comparable weak adhesions after 24 h (Figure 5d). However, compared to the histogram after 2 h, two peaks of FA concentrations were already observed after 24 h; cells that began to attach to the surface (cells with a relatively small number of FAs) and cells with relatively strong adhesions (cells with a high number of FAs (~60)) were found. On the PEEK surface, cells behaved similarly as on the PMMA group: Higher concentrations of cells formed weak adhesions (~20 FAs per cell), but another, relatively lower, portion of cells demonstrated strong attachment (~75–80 per cell) (Figure 5f). The FA numbers in cells grown on Ti-LS2 also increased after 24 h, and the maximum cell concentration was found containing ~25 FAs (Figure 5e). Thus, PEEK and PMMA sample groups induced weak and slow HGF-1 adhesion and comparing FAs/cell, the statistical difference did not exist between these three groups (*p* > 0.05) (Table S5). On the ZrO_2_ surface, cell adhesion during the time also improved: The maximum FA number determined in HGF-1, 24 h after seeding, was ~45 (Figure 5b). Comparing both periods (2 and 24 h), all tested groups showed an increment of FAs, except the Ti alloy surface group. There were no changes in FA numbers on the latter sample, comparing 2 and 24 h.

## 4. Discussion

This study reveals new scope for Ti alloy prosthetic part improvements. Titanium alloys are widespread in implant dentistry and its prosthetics, as well as in orthopedic medicine, because of their relatively bioinert and mechanical properties [31]. Despite that, the usage of metal alloys is limited mainly because of expected corrosion, allergies [32], and aesthetic issues [33]. Moreover, the titanium metal-based surface is sensitive to mechanical damage [34], and the abutment can be scratched during oral hygiene, resulting in retention for plaque accumulation [35]. The latest findings of ceramic prosthetic materials revealed their better bioinertia and peri-implant soft tissue integration [15] and higher surface hardness [36], but their use is relatively limited due to bending sensitivity [37] and brittleness [38,39].

The conventional layering ceramics were successfully applied for porcelain fused to metal restorations for aesthetic reasons. Unfortunately, the fusing on titanium alloys is still quite delicate on a count of lower adhesion strength between the titanium core and ceramic [40]. The weak fusing between these surfaces is mostly related to entrapped sand particles on titanium after sandblasting [41] and thick oxide due to alloy reactivity [42]. Surface improvements by thin films or coatings open up new fields of material improvements and applications. In this study, newly generated bioceramic coatings were expected to improve cell–surface interactions. Similar studies have achieved promising results with coatings of other bioceramics [43,44]. For this study, unique coatings of yttria-stabilized zirconium oxide and lithium disilicate bioceramics using the sol-gel method were selected for surface improvements of a titanium alloy substrate. The bioceramic coating on Ti alloys might be a solution, which allows for combining the mechanical properties of the Ti substrate and the advantages of bioceramics. The substrate isolation by a coating of bioceramics reduces its corrosion capacity and establishes ceramic–tissue contact. The chosen ceramics are widely used for prosthetic purposes, and their biocompatibility and durability have already been proven [45]. The sol-gel method was chosen for coating because of its simplicity, cost-effectiveness, and successful application with other substrates and bioceramic materials [46]. 

By contrast with conventional semi-soft YSZ milling blanks, the high temperature sintering to stabilize the crystalline structure by yttrium was unnecessary in a sol-gel strategy. The crystalline form of zirconium particles is not used for sols-gels, where elements are mixed homogeneously at the molecular level, therefore phase formation temperatures are lower. In this synthesis, the temperature was used for a few reasons: to evaporate solvents, to burn-out the organic components, and to open the pores of the substrate surface for molecules of the coating. There are a number of studies showing that tetragonal YSZ can be obtained from as low as 500 °C [47,48], and Li_2_Si_2_O_5_ at 550 °C [49,50,51]. Therefore, the temperature was lower than for sintering of conventional powder-based YSZ ceramic and was selected regarding the transformation phases of the titanium alloy.

The success of both coatings was confirmed by XRD, which showed chemical deposition of 3YSZ and LS2 on the surface of the polished titanium alloy. Additionally, morphological findings established the distribution of coatings. The layer of 3YSZ was not uniform and showed submicron cracks, whereas the LS2 coatings did not contain any cracking. The nature of the phase composition might influence such behavior: 3YSZ coating is purely crystalline in nature, whereas LS2 generally is glass-ceramic. Therefore, the crystallization mechanism and kinetics are different for these coatings. However, the cracking of the 3YSZ coating could not be related to phase transformation from tetragonal to monoclinic [52], as it was in another study where a different methodology was generated for YSZ coating. In this study, the tetragonal phase was confirmed by XRD, and the only explanation of volume expansion during the cooling might be accepted as a probable factor. It is also important to note that XRD revealed no titanium oxide interlayer; therefore, in both cases, no unwanted oxidation of the Ti alloy substrate took place, which in some cases induces cracking of the upper ceramic film. In fact, this shows that deposited coatings effectively isolate the Ti alloy surface, thus decreasing the probability of releasing allergy-promoting ions such as Al or V.

The AFM was selected as one of the most suitable instruments for describing the surface morphology of biomaterials [53], and the roughness of surface was expressed by a commonly used Sa parameter [54]. Results of surface roughness showed tendencies that additional coatings decreases the roughness by covering and filling remaining scratches and irregularities of the surface. This was statistically confirmed in the Ti-LS2 group (roughness significantly from S_a_ = 17.67 ± 0.35 nm to S_a_ = 9.61 ± 0.66 nm (*p* < 0.0001)), whereas Ti-3YSZ revealed only a slight decrease (to S_a_ = 16.61 ± 0.52 nm (*p* > 0.05)), which could also be related to different crystallization of the coating. Unfortunately, both coatings did not demonstrate such a low surface roughness as on the highly polished ZrO_2_ surface (S_a_ = 5.53 ± 0.21 nm). In this study, the established different roughness between Ti and ZrO_2_ might be related to the origin of the material, and the surface hardness influenced polishing efficiency. The idea of coating might be a prospective possibility to decrease the surface roughness of metal alloys and improve their usage for implant prosthetics and other biomedical implants. However, the thickness of coatings limited their effectiveness to change the substrate-dependent surface roughness.

The experimental coatings used in this study significantly changed the WCA of the surface. After coating, the water contact angle decreased significantly from 60.21 ± 1.58° to 32.58 ± 1.94° in the Ti-3YSZ group and to 25.71 ± 1.33° in the Ti-LS2 group (*p* < 0.0001), thus surfaces became significantly hydrophilic after the deposition of both coatings. Also, in both groups, the total surface free energy increased from 41.81 ± 2.01 mN/m to 60.03 ± 0.57 mN/m in the Ti-3YSZ group and 65.70 ± 1.00 in the Ti-LS2 group (*p* < 0.0001). The polar surface free energy increased from 7.37 ± 1.62 mN/m to 25.14 ± 0.41 mN/m in the Ti-3YSZ group and 33.39 ± 0.59 mN/m in the Ti-LS2 group (*p* < 0.0001). This is be related to changes in surface chemistry and morphology. These coatings are composed of nanosized crystallites that increase the total surface area of the substrate, and in the case of Ti-LS2, Li+ ions were exposed to the surface, which could significantly increase surface polarity as well as hydrophilicity. However, it also must be biocompatible.

The high biocompatibility of experimental coatings was confirmed. It was similar to that of conventional prosthetic materials or even higher and enabled the favorable application of this technology for biomaterials and biomedical applications for future studies. No cytotoxic effects that could be attributed to the new materials and their chemical composition or morphology have been identified. On the other hand, no improvement in biocompatibility was observed with coatings, but it also established the stability of the coatings’ composition. Nevertheless, according to the results of biocompatibility polymeric materials, PEEK and PMMA showed the lowest rates, establishing that these types of materials were less favorable for direct contact with living tissues comparing to inorganic materials. This might be related to the origin of materials and their chemical composition, for example, the remaining free monomer in PMMA [55]. However, it might also be determined by the hydrophobic character of these materials. Thus, materials of inorganic origin exhibited better biocompatibility.

Surface morphology is another relevant factor in determining cell behavior [56]. Also, a negative correlation between the biocompatibility and surface roughness was confirmed (r = −0.7036; *p* < 0.0001) in this study. In all groups with commercially available prosthetic materials, an obvious relationship between the surface roughness and the relative cell count was visible. When the S_a_ decreased, the RCC increased, so the increase of surface smoothness possibly alters the direct interface between the cells and the substrate. By contrast, this tendency was not confirmed with coated surfaces, where the roughness was comparably low. This confirmed that nanoparticles change surface properties as well as interact with living cells [57].

Furthermore, coatings’ improved surface attraction for cell adhesion was also confirmed. The increased surface area occupied by a cell showed the dynamics of cells’ attachment on surfaces. It is known that the faster a cell occupies a larger surface area, the more attractive this substrate is for cell attachment [58]. The largest surface area of HGFs after 2 and 24 h was observed on the Ti-3YSZ surface (1789 μm^2^ and 2630 μm^2^, respectively), so this surface promoted the best and fastest cell adhesion compared to other tested materials. The rapid quantitative cell response and comparable high surface area were also established on Ti-LS2 surface after 2 h: 1507 μm^2^. Unfortunately, the cell expansion on this surface was insignificant after 24 h (1888 μm^2^), despite the lowest roughness and WCA values. However, the qualitative examination of the stained cell on Ti-LS2 after 24 h provided more promising results. The obvious elongation of HGFs and concentrated actin and vinculin at both opposite tails of the spindle was visible. This cellular elongation enables the assumption that on this substrate, cells proliferated faster compared to others [59,60], and because of intensive proliferation, they de-attached from the surface, showing a lower adhesive area as well as a lower amount of FAs. However, for a more comprehensive evaluation, the motility of cells should be observed. Thus, promising results of the adhesive process on the Ti-LS2 substrate were established only after 2 h.

It is interesting that PEEK and PMMA surfaces were less attractive for cells. The results of cell surface area measurements showed that HGF-1 adhesion was slowest on these samples, and the lowest number of formed FAs within the cell was determined in these two polymeric groups. This may be due to the relatively hydrophobic character of surfaces, and hydrophobicity may decrease the attractiveness of surfaces for cell adhesion [61] and soft tissue integration [62].

Cell FA formation during the adhesion process also indicates that the substrate is attractive for cell attachment, and the number of FAs formed in the cell indicates the strength of cell-surface interaction [19,58,63]. In this study, HGF-1, grown on Ti-3YSZ samples (which demonstrated a smooth surface (S_a_ = 16.61 ± 0.52 nm) and hydrophilic character (32.58 ± 1.94° WCA)), formed the highest numbers of FAs compared to other tested specimens after 24 h (66.75 ± 4.91 FAs/cell (*p* ≤ 0.05)). In contrast, FA densities in cells were exceptionally low on polymeric materials, PEEK and PMMA. The latter samples demonstrated the highest surface roughness; thus, the low density of FAs might be a result of that. Other studies have shown that the increase of FAs coincided with decreasing roughness [64,65,66]. On smooth surfaces, a closer cell–substrate contact could be achieved than on rough ones [67]. Furthermore, fibroblasts are rugophobic cells, which means they have adverse reactions to the surface roughness [68]. Moreover, PEEK and PMMA demonstrated more hydrophobic character compared to Ti-3YSZ. Surface hydrophobicity alters the adsorption of extracellular proteins, which in turn could result in weaker FAs [69,70]. Cell attachment on the surface is promoted only during cell response to the extracellular matrix, which is mainly formed by proteins [19]. Therefore, protein adsorption on the surface could be another critical factor for FA formation, and it is closely related to the surface WCA [67].

Thus, surface coatings of titanium alloys allow the physicochemical properties of the surface to be improved, leading to improved behavior of HGFs. Moreover, surface coatings can reduce the accumulation of bacterial plaque, increase surface hardness, and increase the resistance of implant abutment surface scratching [71]. However, these require additional studies. Moreover, the organization of HGFs’ rich connective tissue around a regular titanium surface might be delayed 4-8 weeks at least [6,72,73]. The findings of this study established promising results of 3YSZ and LS2 coatings, which might be useful for in vivo studies for a faster sealing of the peri-implant mucosa. However, the results of this study remain limited, and additional studies of protein adsorption are necessary for a better explanation of cell behavior. Additionally, these novel coatings should be investigated with in vitro bacterial models as well as applied in vivo to determine their influence on clinical outcomes.

## 5. Conclusions

In this study, the novel coatings of 3YSZ and LS2 were achieved by the sol-gel method, while the LS2 coating was used for the first time on titanium alloy. Both of the novel coating materials might be useful for the improvement of the physicochemical properties of titanium alloys. All the investigated materials were biocompatible. The Ti-YSZ was the most suitable for cellular adhesion responses compared to other groups, as this surface demonstrated the most significant cell area (the best cell adhesion) 2630 μm^2^ (*p* ≤ 0.05), and the significantly highest 66.75 ± 4.91 (*p* ≤ 0.05) focal adhesions (FAs) per cell after 24 h post-seeding. PMMA and PEEK showed the worst cellular behavior, demonstrating the highest roughness (S_a_ = 65.23 ± 2.41 nm and S_a_ = 38.00 ± 0.78 nm, respectively, *p* < 0.0001) and WCA (68.84 ± 2.65° and 75.62 ± 2.46°, respectively, *p* < 0.0001). Further studies are needed, but 3YSZ and LS2 coatings might be promising for biomedical applications.

## Figures and Tables

**Table 1:** Descriptive statistics of all groups’ surface physicochemical experiments (mean ± standard deviation): roughness (nm), water contact angle (WCA) (deg.), dispersive surface free energy (SFE_dispersive) (mN/m), polar surface free energy (SFE_polar) (mN/m), total surface free energy (SFE_total) (mN/m). Data of titanium alloy (Ti), yttria-stabilized zirconium oxide coating on titanium alloy (Ti-3YSZ), lithium disilicate coating on titanium alloy (Ti-LS2), yttria-stabilized zirconium oxide, polyether ether ketone composite (PEEK) and poly(methyl methacrylate) (PMMA) groups.

Descriptive Statistics	Ti	Ti-3YSZ	Ti-LS2	ZrO_2_	PEEK	PMMA
Number of values	10	10	10	10	10	10
Roughness	17.67 ± 0.35	16.61 ± 0.52	9.61 ± 0.66	5.53 ± 0.21	38.00 ± 0.78	65.23 ± 2.41
WCA	60.21 ± 1.58	32.58 ± 1.94	25.71 ± 1.33	57.32 ± 1.86	75.62 ± 2.46	68.84 ± 2.65
SFE_dispersive	34.44 ± 1.56	36.89 ± 0.33	32.31 ± 0.59	30.38 ± 0.55	39.32 ± 0.38	36.98 ± 0.59
SFE_polar	7.37 ± 1.62	25.14 ± 0.41	33.39 ± 0.59	14.72 ± 0.36	4.14 ± 0.20	7.36 ± 0.32
SFE_total	41.81 ± 2.01	62.03 ± 0.57	65.70 ± 1.00	45.10 ± 0.34	43.46 ± 0.54	44.34 ± 0.73

**Table 2:** Multiple comparisons of surface roughness (nm) among groups (*n* = 10 for each group) by Tukey’s test and significant difference of means at the *p* ≤ 0.05 level: **** *p* < 0.0001. Non-significant (ns) *p* > 0.05.

Tukey’s Multiple Comparisons Test	Mean 1	Mean 2	Mean Diff.	Adjusted *p* Value
Ti vs. Ti-3YSZ	17.67	16.61	1.062	0.2759 ^ns^
Ti vs. Ti-LS2	17.67	9.61	8.062	<0.0001 ****
Ti vs. ZrO_2_	17.67	5.53	12.14	<0.0001 ****
Ti vs. PEEK	17.67	38.00	20.33	<0.0001 ****
Ti vs. PMMA	17.67	65.23	47.55	<0.0001 ****
Ti-3YSZ vs. Ti-LS2	16.61	9.61	7.00	<0.0001 ****
Ti-3YSZ vs. ZrO_2_	16.61	5.53	11.08	<0.0001 ****
Ti-3YSZ vs. PEEK	16.61	38.00	21.39	<0.0001 ****
Ti-3YSZ vs. PMMA	16.61	65.23	48.61	<0.0001 ****
Ti-LS2 vs. ZrO_2_	9.61	5.53	4.081	<0.0001 ****
Ti-LS2 vs. PEEK	9.61	38.00	28.39	<0.0001 ****
Ti-LS2 vs. PMMA	9.61	65.23	55.61	<0.0001 ****
ZrO_2_ vs. PEEK	5.53	38.00	32.47	<0.0001 ****
ZrO_2_ vs. PMMA	5.53	65.23	59.70	<0.0001 ****
PEEK vs. PMMA	38.00	65.23	27.22	<0.0001 ****

**Table 3:** Multiple comparisons of surface WCA (deg.) among groups (*n* = 10 for each group) by Tukey’s test and significant difference of means at the *p* ≤ 0.05 level: **** *p* < 0.0001; * *p* = 0.0264.

Tukey’s Multiple Comparisons Test	Mean 1	Mean 2	Mean Diff.	Adjusted *p*Value
Ti vs. Ti-3YSZ	60.21	32.58	27.63	<0.0001 ****
Ti vs. Ti-LS2	60.21	25.71	34.50	<0.0001 ****
Ti vs. ZrO_2_	60.21	57.32	2.89	0.0264 *
Ti vs. PEEK	60.21	75.62	15.41	<0.0001 ****
Ti vs. PMMA	60.21	68.84	8.63	<0.0001****
Ti-3YSZ vs. Ti-LS2	32.58	25.71	6.87	<0.0001 ****
Ti-3YSZ vs. ZrO_2_	32.58	57.32	24.74	<0.0001 ****
Ti-3YSZ vs. PEEK	32.58	75.62	43.04	<0.0001 ****
Ti-3YSZ vs. PMMA	32.58	68.84	36.26	<0.0001 ****
Ti-LS2 vs. ZrO_2_	25.71	57.32	31.61	<0.0001 ****
Ti-LS2 vs. PEEK	25.71	75.62	49.91	<0.0001 ****
Ti-LS2 vs. PMMA	25.71	68.84	43.13	<0.0001 ****
ZrO_2_ vs. PEEK	57.32	75.62	18.30	<0.0001 ****
ZrO_2_ vs. PMMA	57.32	68.84	11.52	<0.0001 ****
PEEK vs. PMMA	75.62	68.84	6.778	<0.0001 ****

**Table 4:** Multiple comparisons of surface total SFE (mN/m) among groups (*n* = 10 for each group) by Tukey’s test and significant difference of means at the *p* ≤ 0.05 level: **** *p* < 0.0001; ** *p* = 0.007; **^#^ *p* = 0.0071. Non-significant (ns) *p* > 0.05.

Tukey’s Multiple Comparisons Tests	Mean 1	Mean 2	Mean Diff.	Adjusted *p* Value
Ti vs. Ti-3YSZ	41.81	62.03	20.21	<0.0001 ****
Ti vs. Ti-LS2	41.81	65.70	23.89	<0.0001 ****
Ti vs. ZrO_2_	41.81	45.10	3.29	<0.0001 ****
Ti vs. PEEK	41.81	43.46	1.64	0.0071 **^#^
Ti vs. PMMA	41.81	44.34	2.53	<0.0001 ****
Ti-3YSZ vs. Ti-LS2	62.03	65.70	3.67	<0.0001 ****
Ti-3YSZ vs. ZrO_2_	62.03	45.10	16.93	<0.0001 ****
Ti-3YSZ vs. PEEK	62.03	43.46	18.57	<0.0001 ****
Ti-3YSZ vs. PMMA	62.03	44.34	17.69	<0.0001 ****
Ti-LS2 vs. ZrO_2_	65.70	45.10	20.60	<0.0001 ****
Ti-LS2 vs. PEEK	65.70	43.46	22.24	<0.0001 ****
Ti-LS2 vs. PMMA	65.70	44.34	21.36	<0.0001 ****
ZrO_2_ vs. PEEK	45.10	43.46	1.64	0.007 **
ZrO_2_ vs. PMMA	45.10	44.34	0.76	0.5408 ^ns^
PEEK vs. PMMA	43.46	44.34	0.88	0.37 ^ns^

**Table 5:** Descriptive statistics of all groups’ focal adhesions (FAs) per cell after 2 and 24 h (mean ± standard error of mean).

Descriptive Statistics	Ti	Ti-3YSZ	Ti-LS2	ZrO_2_	PEEK	PMMA
FAs per cell (2 h)	36.16 ± 2.79	28.00 ± 3.76	16.94 ± 1.57	29.02 ± 2.42	5.15 ± 0.74	5.76 ± 0.93
FAs per cell (24 h)	43.47 ± 3.14	66.75 ± 4.91	26.03 ± 2.98	47.69 ± 3.27	23.45 ± 3.71	28.66 ± 3.67

## Supplementary Materials

The following are available online at https://www.mdpi.com/1996-1944/13/9/2070/s1, Table S1: Multiple comparisons of surface polar SFE (mN/m) among groups (n = 10 for each group) by Tukey’s test and significant difference of means at the *p* ≤ 0.05 level: **** *p* < 0.0001.; Table S2. Multiple comparisons of surface dispersive SFE (mN/m) among groups (n = 10 for each group) by Tukey’s test and significant difference of means at the *p* ≤ 0.05 level: **** *p* < 0.0001.; Table S3. Pearson correlation between experiments (n = 60 for each experiment) significant at the *p* ≤ 0.05 level: **** *p* < 0.0001; *** *p* = 0.0008; * *p* = 0.0497. Non-significant (ns) *p* value of correlation > 0.05.; Table S4. Multiple comparisons of focal adhesions (FAs) per cell after 2 h among groups by Tukey’s test and significant difference of means at the *p* ≤ 0.05 level: **** *p* < 0.0001; **# *p* = 0.0012; #** *p* = 0.0014; #**# *p* = 0.0022; ** *p* = 0.0059.; Table S5. Multiple comparisons of focal adhesions (FAs) per cell after 24 h among groups by Tukey’s test and significant difference of means at the *p* ≤ 0.05 level: **** *p* < 0.0001; *** *p* = 0.0003; **# *p* = 0.0011; #** *p* = 0.0016; #**# *p* = 0.0027; ** *p* = 0.0067.
