# Supplementary material for: Novel Yttria-Stabilized Zirconium Oxide and Lithium Disilicate Coatings on Titanium Alloy Substrate for Implant Abutments and Biomedical Application
Source: Materials (Basel). 2020 Apr 30;13(9):2070. doi: 10.3390/ma13092070 (PMC7254192; doi:10.3390/ma13092070)
Supplement: Supplementary file 1 [file materials-13-02070-s001.pdf]

materials

Supplementary Materials

# Novel Yttria-Stabilized Zirconium Oxide and Lithium Disilicate Coatings on Titanium Alloy Substrate for Implant Abutments and Biomedical Application

**Julius Maminskas [1,*], Jurgis Pilipavicius [2,3], Edvinas Staisiunas [3], Gytis Baranovas [3], Milda Alksne [4], Povilas Daugela [5] and Gintaras Juodzbalys [5]**

The results of statistical data analysis.

**Table S1.** Multiple comparisons of surface polar SFE (mN/m) among groups (n = 10 for each group) by Tukey's test and significant difference of means at the $p \leq 0.05$ level: **** $p < 0.0001$. Non-significant (ns) – $p > 0.05$.

| Tukey's Multiple Comparisons Tests | Mean 1 | Mean 2 | Mean Diff, | Adjusted P Value |
|---|---|---|---|---|
| Ti vs. Ti-3YSZ | 7.37 | 25.14 | 17.77 | <0.0001**** |
| Ti vs. Ti-LS2 | 7.37 | 33.39 | 26.02 | <0.0001**** |
| Ti vs. $ZrO_2$ | 7.37 | 14.72 | 7.35 | <0.0001**** |
| Ti vs. PEEK | 7.37 | 4.14 | 3.23 | <0.0001**** |
| Ti vs. PMMA | 7.37 | 7.36 | 0.01 | >0.9999 ns |
| Ti-3YSZ vs. Ti-LS2 | 25.14 | 33.39 | 8.25 | <0.0001**** |
| Ti-3YSZ vs. $ZrO_2$ | 25.14 | 14.72 | 10.42 | <0.0001**** |
| Ti-3YSZ vs. PEEK | 25.14 | 4.14 | 21.00 | <0.0001**** |
| Ti-3YSZ vs. PMMA | 25.14 | 7.36 | 17.78 | <0.0001**** |
| Ti-LS2 vs. $ZrO_2$ | 33.39 | 14.72 | 18.67 | <0.0001**** |
| Ti-LS2 vs. PEEK | 33.39 | 4.14 | 29.25 | <0.0001**** |
| Ti-LS2 vs. PMMA | 33.39 | 7.36 | 26.03 | <0.0001**** |
| $ZrO_2$ vs. PEEK | 14.72 | 4.14 | 10.58 | <0.0001**** |
| $ZrO_2$ vs. PMMA | 14.72 | 7.36 | 7.36 | <0.0001**** |
| PEEK vs. PMMA | 4.14 | 7.36 | 3.22 | <0.0001**** |

**Table S2.** Multiple comparisons of surface dispersive SFE (mN/m) among groups (n = 10 for each group) by Tukey's test and significant difference of means at the $p \leq 0.05$ level: **** $p < 0.0001$. Non-significant (ns) – $p > 0.05$.

| Tukey's Multiple Comparisons Tests | Mean 1 | Mean 2 | Mean Diff, | Adjusted P Value |
|---|---|---|---|---|
| Ti vs. Ti-3YSZ | 34.44 | 36.89 | 2.45 | <0.0001**** |
| Ti vs. Ti-LS2 | 34.44 | 32.31 | 2.13 | <0.0001**** |
| Ti vs. $ZrO_2$ | 34.44 | 30.38 | 4.06 | <0.0001**** |
| Ti vs. PEEK | 34.44 | 39.32 | 4.88 | <0.0001**** |
| Ti vs. PMMA | 34.44 | 36.98 | 2.54 | <0.0001**** |
| Ti-3YSZ vs. Ti-LS2 | 36.89 | 32.31 | 4.58 | <0.0001**** |
| Ti-3YSZ vs. $ZrO_2$ | 36.89 | 30.38 | 6.51 | <0.0001**** |

| | | | | |
|---|---|---|---|---|
| Ti-3YSZ vs. PEEK | 36.89 | 39.32 | 2.43 | <0.0001**** |
| Ti-3YSZ vs. PMMA | 36.89 | 36.98 | 0.09 | 0.9998$^{ns}$ |
| Ti-LS2 vs. $ZrO_2$ | 32.31 | 30.38 | 1.93 | <0.0001**** |
| Ti-LS2 vs. PEEK | 32.31 | 39.32 | 7.01 | <0.0001**** |
| Ti-LS2 vs. PMMA | 32.31 | 36.98 | 4.67 | <0.0001**** |
| $ZrO_2$ vs. PEEK | 30.38 | 39.32 | 8.94 | <0.0001**** |
| $ZrO_2$ vs. PMMA | 30.38 | 36.98 | 6.60 | <0.0001**** |
| PEEK vs. PMMA | 39.32 | 36.98 | 2.34 | <0.0001**** |

**Table S3.** Pearson correlation between experiments (n = 60 for each experiment) significant at the $p \leq 0.05$ level: **** $p < 0.0001$; *** $p = 0.0008$; * $p = 0.0497$. Non-significant (ns) $p$ value of correlation >0.05. Non-significant (ns) – $p > 0.05$.

| **Pearson Correlation** | **Roughness vs. WCA** | **Roughness vs. SFE_tot** | **Roughness vs. Biocompatibility** |
|---|---|---|---|
| r | 0.6127 | –0.4199 | –0.7036 |
| R squared | 0.3753 | 0.1764 | 0.4951 |
| P (two-tailed) | <0.0001**** | 0.0008*** | <0.0001**** |
| Pearson correlation | WCA vs. SFE_tot | WCA vs. Biocompatibility | SFE_tot vs. Biocompatibility |
| r | –0.9348 | –0.2545 | 0.06113 |
| R squared | 0.8739 | 0.06479 | 0.003737 |
| P (two-tailed) | <0.0001**** | 0.0497* | 0.6427$^{ns}$ |

**Table S4.** Multiple comparisons of focal adhesions (FAs) per cell after 2 h among groups by Tukey's test and significant difference of means at the $p \leq 0.05$ level: **** $p < 0.0001$; $^{**\#}$ $p = 0.0012$; $^{\#**}$ $p = 0.0014$; $^{\#**\#}$ $p = 0.0022$; ** $p = 0.0059$.

| **Tukey's Multiple Comparisons Tests** | **Mean 1** | **Mean 2** | **Mean Diff** | **Adjusted P Value** |
|---|---|---|---|---|
| Ti vs. Ti-3YSZ | 36.16 | 28.00 | 8.16 | 0.112$^{ns}$ |
| Ti vs. Ti-LS2 | 36.16 | 16.94 | 19.22 | <0.0001**** |
| Ti vs. $ZrO_2$ | 36.16 | 29.02 | 7.14 | 0.2002$^{ns}$ |
| Ti vs. PEEK | 36.16 | 5.15 | 31.00 | <0.0001**** |
| Ti vs. PMMA | 36.16 | 5.76 | 30.39 | <0.0001**** |
| Ti-3YSZ vs. Ti-LS2 | 28.00 | 16.94 | 11.06 | 0.0059** |
| Ti-3YSZ vs. $ZrO_2$ | 28.00 | 29.02 | 1.02 | 0.9995$^{ns}$ |
| Ti-3YSZ vs. PEEK | 28.00 | 5.15 | 22.85 | <0.0001**** |
| Ti-3YSZ vs. PMMA | 28.00 | 5.76 | 22.24 | <0.0001**** |
| Ti-LS2 vs. $ZrO_2$ | 16.94 | 29.02 | 12.08 | 0.0012$^{**\#}$ |
| Ti-LS2 vs. PEEK | 16.94 | 5.15 | 11.79 | 0.0014$^{\#**}$ |
| Ti-LS2 vs. PMMA | 16.94 | 5.76 | 11.18 | 0.0022$^{\#**\#}$ |
| $ZrO_2$ vs. PEEK | 29.02 | 5.15 | 23.86 | <0.0001**** |
| $ZrO_2$ vs. PMMA | 29.02 | 5.76 | 23.25 | <0.0001**** |
| PEEK vs. PMMA | 5.15 | 5.76 | 0.61 | >0.9999$^{ns}$ |

**Table S5.** Multiple comparisons of focal adhesions (FAs) per cell after 24 h among groups by Tukey's test and significant difference of means at the $p \leq 0.05$ level: **** $p < 0.0001$; *** $p = 0.0003$; **# $p = 0.0011$; #** $p = 0.0016$; #**# $p = 0.0027$; ** $p = 0.0067$. Non-significant (ns) – $p > 0.05$.

| Tukey's Multiple Comparisons Tests | Mean 1 | Mean 2 | Mean Diff, | Adjusted P Value |
|---|---|---|---|---|
| Ti vs. Ti-3YSZ | 42.23 | 66.75 | 24.52 | 0.0003*** |
| Ti vs. Ti-LS2 | 42.23 | 26.03 | 16.20 | 0.0753 ns |
| Ti vs. $ZrO_2$ | 42.23 | 47.69 | 5.46 | 0.9029ns |
| Ti vs. PEEK | 42.23 | 23.45 | 18.78 | 0.0067** |
| Ti vs. PMMA | 42.23 | 28.66 | 13.57 | 0.1339 ns |
| Ti-3YSZ vs. Ti-LS2 | 66.75 | 26.03 | 0.72 | <0.0001**** |
| Ti-3YSZ vs. $ZrO_2$ | 66.75 | 47.69 | 19.06 | 0.0027#**# |
| Ti-3YSZ vs. PEEK | 66.75 | 23.45 | 43.3 | <0.0001**** |
| Ti-3YSZ vs. PMMA | 66.75 | 28.66 | 38.09 | <0.0001**** |
| Ti-LS2 vs. $ZrO_2$ | 26.03 | 47.69 | 21.66 | 0.0011**# |
| Ti-LS2 vs. PEEK | 26.03 | 23.45 | 2.58 | 0.9972 ns |
| Ti-LS2 vs. PMMA | 26.03 | 28.66 | 2.63 | 0.9972 ns |
| $ZrO_2$ vs. PEEK | 47.69 | 23.45 | 24.25 | <0.0001**** |
| $ZrO_2$ vs. PMMA | 47.69 | 28.66 | 19.03 | 0.0016#** |
| PEEK vs. PMMA | 23.45 | 28.66 | 5.21 | 0.9019 ns |

© 2020 by the authors. Submitted for possible open access publication under the terms and conditions of the Creative Commons Attribution (CC BY) license (http://creativecommons.org/licenses/by/4.0/).
